# RUNX3 loss turns on the dark side of TGF-beta signaling

**DOI:** 10.18632/oncoscience.382

**Published:** 2017-11-28

**Authors:** Vaidehi Krishnan, Yoshiaki Ito

**Affiliations:** Cancer Science Institute of Singapore, National University of Singapore, Singapore-117599

**Keywords:** TGF-beta, RUNX3, HMOX1, DNA damage

The cytokine TGF-β is well-known to play the “Jekyll and Hyde” with cancer cells [1]. On one hand, TGF-β signaling prevents carcinogenesis in early- stage certain cancers by mediating cell cycle-inhibition and apoptosis. On the other hand, TGF-β promotes carcinogenesis in late-stage cancers by inducing invasion, migration and metastasis, partly by the induction of epithelial-to-mesenchymal transition (EMT). Understanding the factors that determine whether TGF-β engages in tumor suppression or tumor promotion has remained a subject of intrigue and clinical interest.

In this regard, earlier studies have shown that the RUNX family of proteins influence TGF-β signaling through multiple mechanisms. The RUNX genes, *RUNX1* and *RUNX3* in particular, are frequently inactivated in human cancers at different stages of carcinogenesis [2]. The RUNX proteins are multifunctional and protect cells from transformation by regulating WNT, Ras-ERK, YAP, BMP, Notch, Mitosis, DNA repair and TGF-β in a contextual manner [2]. Mechanistically, RUNX proteins control these diverse tumor-suppressive networks either by transcriptional regulation via canonical DNA-binding or by non-transcriptional mechanisms.

Historically, the co-operation between the TGF-β signaling and RUNX proteins was discovered during the study of immunoglobulin (IgA) transcription in B lymphocytes. RUNX proteins were shown to physically interact with SMADs, the molecular workhorses of the TGF-β pathway to regulate immunoglobulin transcription. Along similar lines, RUNX proteins together with the SMADs regulate TGF-β-dependent transcription of the cycle inhibitor, p21, and the apoptosis inducer, Bim. Hence, epithelial cells derived from RUNX3-deficient mice were impaired for p21 and Bim expression and displayed spontaneous EMT [3-5]. In the above-mentioned cellular contexts, *RUNX3* deficiency dampens the tumor- suppressive arm of the TGF-β signaling pathway.

In our recent work, we have uncovered that the loss of *RUNX3* sways TGF-β signaling towards tumor promotion [6]. Utilising a non-small cell lung cancer model of TGF-β-mediated EMT, we found that the loss of *RUNX3* promoted oxidative DNA damage when exposed to exogenous TGF-β. TGF-β is known to stimulate ROS production mainly through elevated SMAD-dependent pro-oxidant *NOX4* expression. In our mechanistic studies, RUNX3 counteracted TGF-β-dependent ROS accumulation by upregulation of a redox regulator, Heme oxygenase 1 (*HMOX1* or *HO-1*). HMOX1 is a metabolic enzyme that catalyzes the production of bilirubin, a potent anti-oxidant. The oxidative-DNA damage that accompanied the loss of *RUNX3*, in turn, triggered cellular senescence accompanied by the expression of inflammatory cytokine and chemokines, called as the senescence-associated secretory phenotype (SASP). Of note, increased SASP has recently assumed a clinical relevance given its ability to amplify carcinogenesis in a paracrine manner [7]. Consistently, lung adenocarcinomas harbouring concurrent TGF-β gene expression signature with *RUNX3* loss displayed higher levels of genomic instability and poorer survival. In other words, RUNX3 deficiency augments the tumor-promoting arm of the TGF-β signaling pathway by exacerbating DNA damage and genomic instability.

**Figure 1:**
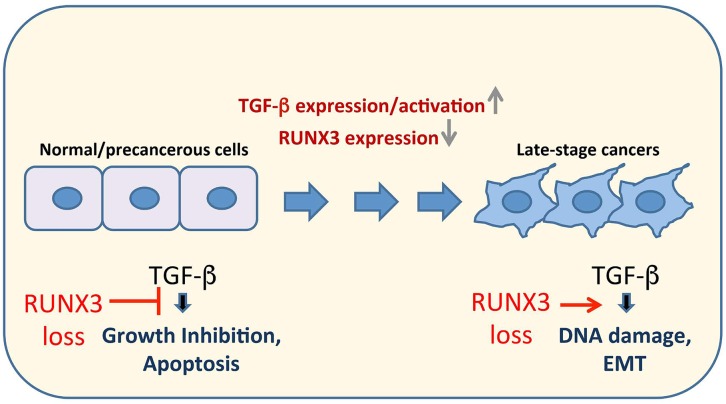
A model on how *RUNX3* loss promotes the pro-carcinogenic functions of TGF-β signaling

Taken together, our study exemplifies how the TGF-β signaling pathway is rendered more tumorigenic upon the loss of *RUNX3* (Figure 1). The induction of genomic instability in a cell-extrinsic manner is perhaps another ill-consequence of pro-carcinogenic TGF-β signaling. Second, RUNX3 protects genomic integrity through *HMOX1* transcriptional regulation although the underlying molecular basis needs future studies. Third, similar to RUNX3, lower RUNX1-induced DNA damage accumulation in the presence of TGF-β, indicating a conservation of function within this family of transcription factors. Fourth, the DNA double strand breaks generated by loss of *RUNX3* triggered cellular senescence upon TGF-β exposure in an ATM- and ATR-dependent manner. Thus, TGF-β-elicited cell fate can be modulated by DNA damage response (DDR) kinases. Lastly, the findings are consistent with our earlier study on the role of RUNX1 and RUNX3 as regulators of DNA repair in a non- transcriptional manner. By facilitating the recruitment of DNA repair protein FANCD2 to sites of damage, RUNX proteins were shown to regulate the Fanconi anemia pathway of DNA repair [8]. It is plausible that the RUNX proteins regulate a larger repertoire of DNA repair processes, emphasising their role as unique tumor suppressors with genome maintenance function.

In conclusion, the complexities underlying TGF-β signalling present a challenge; but these complexities can be converted into a therapeutic opportunity. Based on our studies and work from others, RUNX3 constitutes at least one important node that determines whether TGF-β operates as Jekyll or Hyde in cancers. Manipulating genetic networks downstream of RUNX3 can perhaps swing the TGF-β signaling pendulum from tumor promotion to tumor suppression.
